# Tunable optical filters with wide wavelength range based on porous multilayers

**DOI:** 10.1186/1556-276X-9-427

**Published:** 2014-08-22

**Authors:** Ulrich Mescheder, Isman Khazi, Andras Kovacs, Alexey Ivanov

**Affiliations:** 1 Institute for Applied Research and Faculty of Medical and Mechanical Engineering, Furtwangen University, Robert-Gerwig-Platz 1, 78120 Furtwangen, Germany

**Keywords:** Tunable optical filter, Porous silicon, Photonic crystal, Dual tunability, Silicon anodization, MEMS, MOEMS, Biosensor

## Abstract

A novel concept for micromechanical tunable optical filter (TOF) with porous-silicon-based photonic crystals which provide wavelength tuning of *ca*. ±20% around a working wavelength at frequencies up to kilohertz is presented. The combination of fast mechanical tilting and pore-filling of the porous silicon multilayer structure increases the tunable range to more than 200 nm or provides fine adjustment of working wavelength of the TOF. Experimental and optical simulation data for the visible and near-infrared wavelength range supporting the approach are shown. TOF are used in spectroscopic applications, e.g., for process analysis.

## Background

Tunable optical filter (TOF) is used in spectroscopic applications e.g., for process analyses. Over the last few years, research has been focusing on miniaturizing TOF for applications in microoptical electromechanical systems (MOEMS). For example, TOF systems based on MOEMS Fabry-Perot interferometers (FPI) have been reported, where wavelength tuning results from changing the gap between the involved mirrors and thus requires an extremely precise control of the micromechanical movement [1-4]. In [5] a system with thermal actuation for changing the refractive medium inside the FPI was presented, which provides relatively small tuning range and low frequency response. A tunable optical filter using porous silicon and sub-surface electropolishing was developed by Lammel et al. [6]. In that work, the flip-up optical filter was tilted and tuned by two sophisticated thermal bimorph microactuators where tilt position could not be controlled exactly. Change of spectral response of photonic crystals based on porous multilayers using pore-filling, including fabrication and characterization aspects, and application of this method for sensing were reported by different research groups [7-10]. In a similar approach, Ruminski et al. [11] demonstrated spectral wavelength shifts of porous-silicon-based photonic crystals due to tilting and irreversible pore-filling with polystyrene as optical reference.

In our paper an approach for a tunable micromechanical TOF system based on porous silicon 1D photonic crystal is presented. This MOEMS TOF system, in contrast to the above mentioned examples, can be tuned over a wide wavelength range based on a dual tuning principle: by tilting the photonic crystal and by reversible filling the pores of the photonic crystal with liquids or gases.

Porous-silicon-based 1D photonic crystals forming Bragg filters, rugate filters, microcavities, or other optical components show a pronounced resonant peak of the stop band or a sharp resonant fall-off within the stop band. For a distributed Bragg reflector (DBR) with layers of alternating high and low refractive indices *n*_L_ and *n*_H_, the position of the resonance peak (central wavelength *λ*_0_) is given by

(1) $$n_{H\;}d_{H}=\;\frac{\lambda_{0}}{4}=\;n_{L}d_{L}$$

where *d*_L_ and *d*_H_ are the thicknesses of low and high refractive index layers, respectively. The bandwidth (Δ*λ*) of the so-called stop band around the central wavelength (*λ*_0_) can be selected by the proper adjustment of *n*_L_ and *n*_H_ and is given for DBR by [12]

(2) $$\frac{\Delta \lambda }{\lambda_{0}}=\frac{4}{\pi }\cdot {\text{sin}}^{-1}\frac{n_{H}-n_{L}}{n_{H}+n_{L}}$$

The shift of the central wavelength *λ*_0_ in the transmission or reflection spectrum as function of incidence angle (θ ) can be described with the Bragg's law [6]:

(3) $$\lambda_{\theta }=\lambda_{0}\sqrt{1-{\left(\frac{\text{sin}\theta }{n}\;\right)}^{2}}$$

(4) $$\lambda_{0}=2\mathrm{dn}$$

where *d* is the thickness of a period of the two layers with low and high refractive indices (*d* = *d*_L_ + *d*_H_), and *n* is the effective refractive index of the porous layer.

According to Equation 3, fast tuning of some hundreds of nanometers to shorter wavelengths (blue shift) of the resonant peak position can be achieved by a relatively large rotation (up to 20° to 40°) of the photonic crystal in respect to the incident light.

By pore-filling of the porous optical filter with different gases or liquids (organic or aqueous solutions), shift to longer wavelengths (red shift) of the central wavelength can be achieved. This shift is due to increase of the effective refractive index of the porous silicon during pore-filling. It is important to note that the response times for this tuning principle are limited by the transport processes in nanostructured layers [13].

## Methods

The photonic crystals used for the demonstration of tuning principles in this paper have been fabricated from p-type boron-doped one-side-polished silicon wafers (10 to 20 Ω cm). The backside (not polished side) was doped additionally with boron by ion implantation to achieve low sheet resistance about 24 Ω/□ in order to provide good electrical contact of the wafer's backside to the electrolyte during the anodization process. Silicon samples have been processed from 4-in. wafers by cleaving the wafers to quarters. The area exposed to the electrolyte was 28 × 28 mm^2^. The samples were anodized at room temperature in a double-tank cell (AMMT GmbH, Frankenthal, Germany) with two platinum electrodes operated under current control. Electrolyte mixture of 1:1 volume ratio of 50 wt.% HF and pure ethanol was used. Two types of photonic crystals were realized - DBR and rugate filters. The DBR filters comprised 20 porous layers with alternating low and high refractive indices. The rugate filters were fabricated by sinusoidal modulation of refractive index with 16 and 32 periods. The time-dependent current profiles for anodization were calculated based on experimentally determined dependencies on current density of the effective refractive index (calculated using the Bruggeman model [8] from porosity values) and of porous silicon formation rate. The power supply for the anodization process was provided with NI LabView™ controlled Gossen Metrawatt PSP-1500 power source (Gossen Metrawatt, Nürnberg, Germany). The current density for all filters fabricated in this work was set between 20 and 70 mA/cm^2^. All photonic crystals were designed and fabricated to have a central wavelength *λ*_0_ in the visible spectrum.

**Figure 1:**
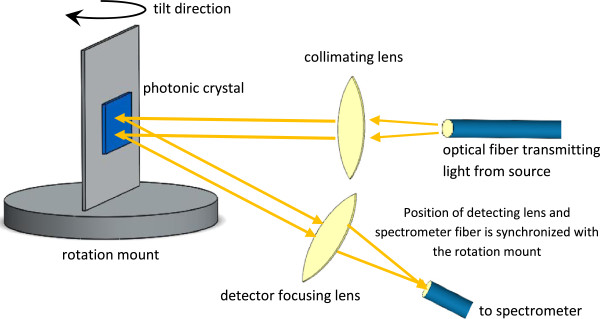
Optical setup for measurements of the spectrum of a photonic crystal at various tilting angles.

An optical setup was constructed in order to measure the tunability induced by tilting and pore-filling of a photonic crystal (Figure 1). The setup consisted of a halogen lamp (12 V, 50 W) emitting light in the visible and near-infrared region, an Avantes FC-UV400-1-ME (Avantes, Apeldoorn, the Netherlands) optical patch cable guiding the light from the halogen lamp towards the porous silicon photonic crystal, a plano-convex lens to collimate the diverging light beam from the optical fiber patch cable, a manual rotation mount with 360° angle of rotation with minimum precision of 2° angle of rotation, and finally an AvaSpec-2048 spectrometer (Avantes). Light reflected from the photonic crystal was guided to the spectrometer by a fiber. The entire setup was assembled on an optical breadboard with all components being firmly fixed to avoid vibrations. The photonic crystal was attached to a holder which was fixed on the rotational mount. Normal incidence of the collimated light on the photonic crystal was chosen. The AvaSpec-2048 spectrometer input fiber was fixed on another optical sliding rail with its position synchronized with the angle of rotation mount. To measure the influence of tilting the photonic crystal on the shift of the central wavelength to *λ*_*θ*_, the rotational mount was rotated manually from 0° (normal incidence) to 50° in steps of 10°.

In order to measure the dual tunability with the pore-filling and the tilting, a closed chamber with dedicated inlet and outlet orifices for vapor or liquid, an anti-reflection glass window, and a holder for the porous Si photonic crystal was constructed. Ethanol vapor was pumped into the closed chamber by a self-designed circulating system through the inlet orifice and left through the outlet orifice. The spectrometer detector fiber was synchronized to the rotation in such a way that this detector fiber was always aligned to the light reflected from the crystal. In order to characterize the dual tunability, the spectrum of the photonic crystal was measured for each tilting angle for two states. First, the spectrum of the photonic crystal in the empty chamber (pores filled with air) was recorded. Afterwards, the chamber was filled with vapor, which resulted in capillary condensation of vapor in the pores of the photonic crystal. Then the spectrum was recorded again.

## Results

**Figure 2:**
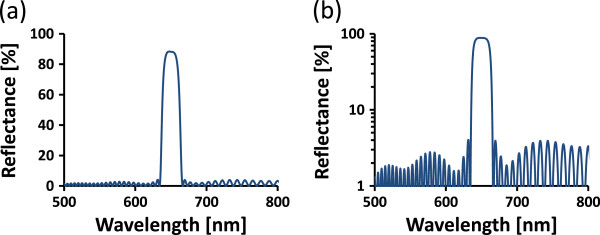
**Simulated spectrum for incident normal light beam.** Simulated spectrum of rugate filter with apodization and index matching, with narrow peak, suppressed side lobes, and suppressed higher-order harmonics: **(a)** with the vertical axis in linear scale and **(b)** with the vertical axis in logarithmic scale.

Essential Macleod software was used to simulate optical properties of the used multilayer structures. The influence of fabrication conditions with varying parameters such as modulating refractive indices and the number of used layers on the reflectance spectrum was investigated. The DBR stack of dielectric multilayers with alternating low and high refractive indices *n*_H_ and *n*_L_ and individual layer thickness values *d*_H_ and *d*_L_ fulfilling the quarter wavelength condition has been simulated for a central wavelength at 650 nm. Rugate filters were simulated with periodic, continuous transition between the low and high refractive indices, resulting in a narrow stop band gap. The application of apodization to the rugate filters [14] resulted in suppression of side lobes and index matching at the multilayer boundary, i.e., air and silicon substrate resulted in suppression of higher order harmonics. As an example, the resulting simulated spectrum for incident normal light beam (0°) is shown in Figure 2.

**Figure 3:**
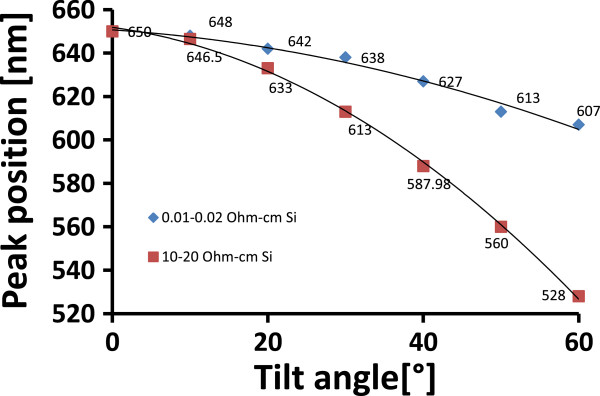
**Comparison of simulated shift of the central wavelength for low-doped and high-doped silicon photonic crystals.** Comparison of simulated shift of the central wavelength due to tilting for high-doped (0.01 to 0.02 Ω cm) and low-doped (10 to 20 Ω cm) porous-silicon-based 1D photonic crystals.

In order to simulate the tunability induced by tilting the photonic crystal, a DBR photonic crystal with 20 layers was designed with a central wavelength *λ*_0_ at 650 nm. Tunability induced by tilting the photonic crystal was simulated for both high-doped (0.01 to 0.02 Ω cm) and low-doped (10 to 20 Ω cm) conditions. The plot of the position of the central wavelength as a function of the tilt angle is shown in Figure 3.

**Figure 4:**
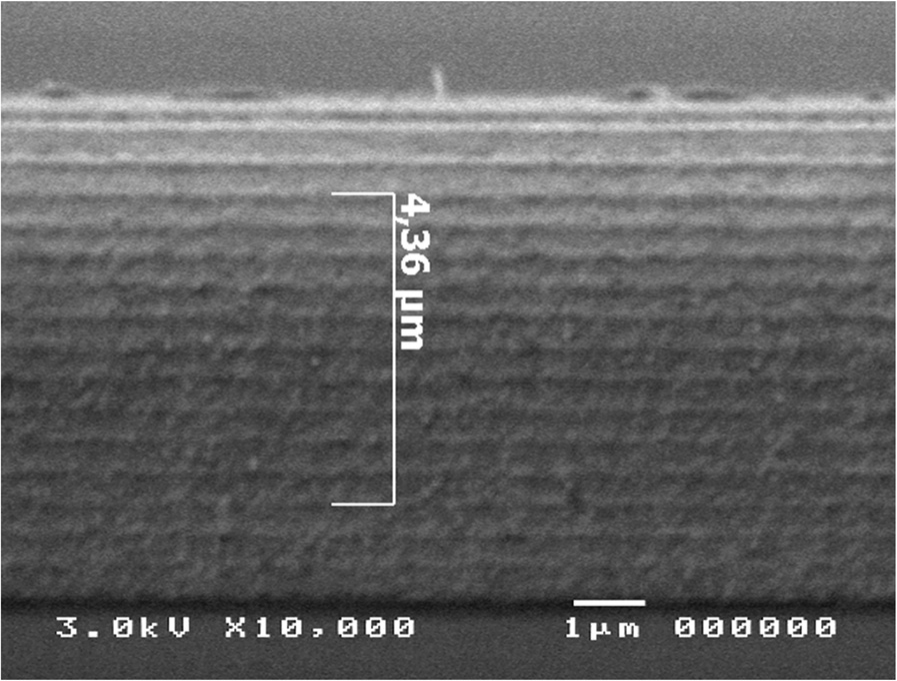
**SEM cross section of the fabricated porous-silicon-based DBR photonic crystal.** SEM cross section of the fabricated porous-silicon-based DBR photonic crystal with alternating low and high refractive indices *n*_H_ and *n*_L_ with individual layer thickness values *d*_H_ and *d*_L_ corresponding to the quarter wave condition.

**Figure 5:**
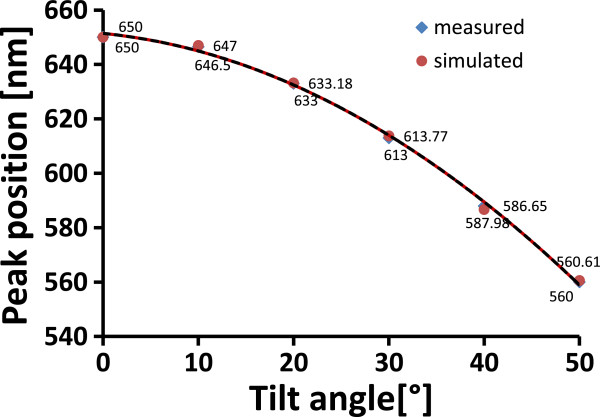
Comparison of the simulated and experimental results for tilting the photonic crystal.

**Figure 6:**
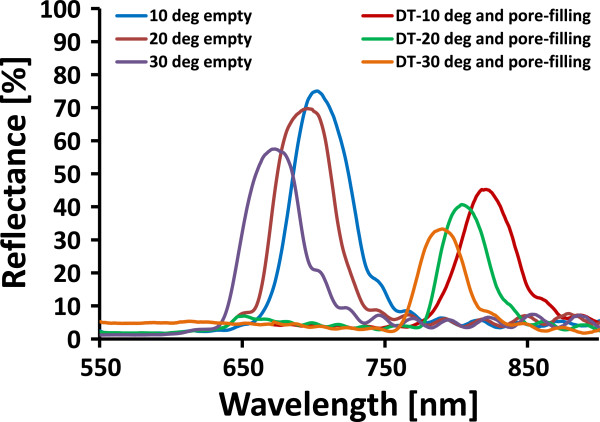
**Experimental measured spectra for dual tunability.** The central wavelength shift in the left part of the plot is due to tilting the photonic crystal up to 30°. The central wavelength shift in the right side of the plot is due to the dual tuning by both tilting and pore-filling of the photonic crystal.

To measure experimentally the tunability induced by tilting, the DBR photonic crystal with refractive index contrast $\frac{n_{H\;}}{n_{L}}=1.1423$ and central wavelength at 650 nm fabricated from the low-doped p-type silicon was used. A scanning electron microscope (SEM) image (cross section through such a DBR) is shown in Figure 4. The measured shift of the central wavelength as a function of the tilt angle is shown in Figure 5.Measurements for demonstration of the dual tunability induced by tilting and pore-filling were performed using a rugate photonic crystal having 32 periods and a central wavelength at 700 nm. As expected from simulation, a small blue shift as a result of tilting and a wider red shift as a result of pore-filling were observed (Figure 6).

## Discussion

From the simulation (Figure 3) and the experimental results (Figure 5), it is clearly demonstrated that tilting the photonic crystal causes a shift of the central wavelength to a lower wavelength, i.e., a blue shift of the spectrum. The tunability range of a low-doped porous silicon photonic crystal by tilting was found to be wider than that of the high-doped photonic crystal (Figure 3). This effect can be explained by a difference in refractive index contrast *n*_H_/*n*_L_ for the two doping levels, where the low-doped porous silicon photonic crystal has a lower refractive index contrast. The measured spectral shift of the central wavelength as function of tilt angle for the low-doped photonic crystal was found to be in good agreement with the simulation (Figure 5). The experiment showed that the shift of the central wavelength as a result of tilting is instantaneous without any noticeable delay. Tunability by the tilting worked well in a narrow wavelength range limited by tilting angles up to 50°. For higher tilting angles, the integrity of the spectrum tended to fade away due to total internal reflection.

**Figure 7:**
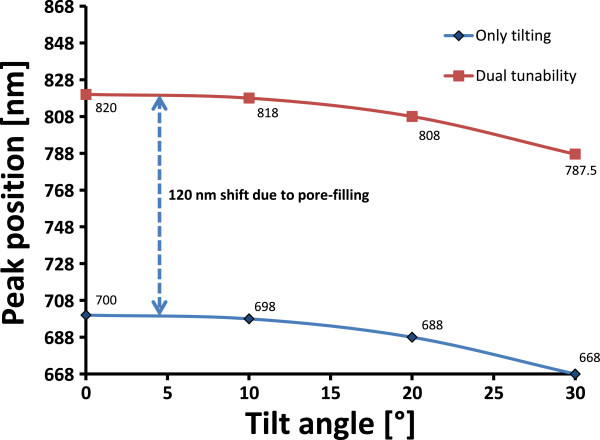
Measured shift of the central wavelength in case of tilting and pore-filling.

When the photonic crystal is filled with ethanol vapor, the capillary condensation within the mesoporous layers (pore size of some nanometers) of the photonic crystal occurs and changes the refractive index contrast thereby shifting the central wavelength to a higher wavelength (red shift). The shift of the central wavelength due to pore-filling is higher than the shift resulting due to the tilting. It was also observed that spectral shift due to pore-filling is not instantaneous but has a delay of few seconds depending on how quick the pores are filled with ethanol vapor. As shown in Figure 6, the central wavelength shift in the left part of the plot is due to the tilting the photonic crystal up to 30°. The central wavelength shift in the right side of the plot is due to the dual tuning by both tilting and pore-filling of the photonic crystal. For example, tilting of the photonic crystal by 10° and using a working wavelength of *λ*_0_ = 700 nm resulted in a shift of *λ*_0_ to a lower wavelength *λ*_*θ*_ = 698 nm. Filling of the pores of the photonic crystal at this tilted position resulted in a shift towards higher wavelength (e.g., at 818 nm). The shift of the central wavelength due to pore-filling is 120 nm for all applied tilting angles, i.e., the gradient of the central wavelength shift due to tilting is the same for the empty and pore-filled photonic crystal as shown in Figure 7. However, in the case of the pore-filling the reflectance intensity of the central wavelength decreased at the shifted wavelength position as the photonic crystal was optimized for air but not for the pore-filled state. Altogether, the dual tunability provided tuning of the central wavelength in both directions of the measured spectrum approximately 20% around the central wavelength.

### System concept

A concept of miniaturized MOEMS system with the integration of both tuning principles has been developed. The tilting angle of photonic crystals is limited by the phenomenon of total internal reflection; therefore, angles up to 20° to 40° are required from the system. For a miniaturized actuation system, this tilting range is challenging. Various actuation principles for tilting such as electrostatic, electromagnetic, piezoelectric, and thermoelectric have been evaluated.

**Figure 8:**
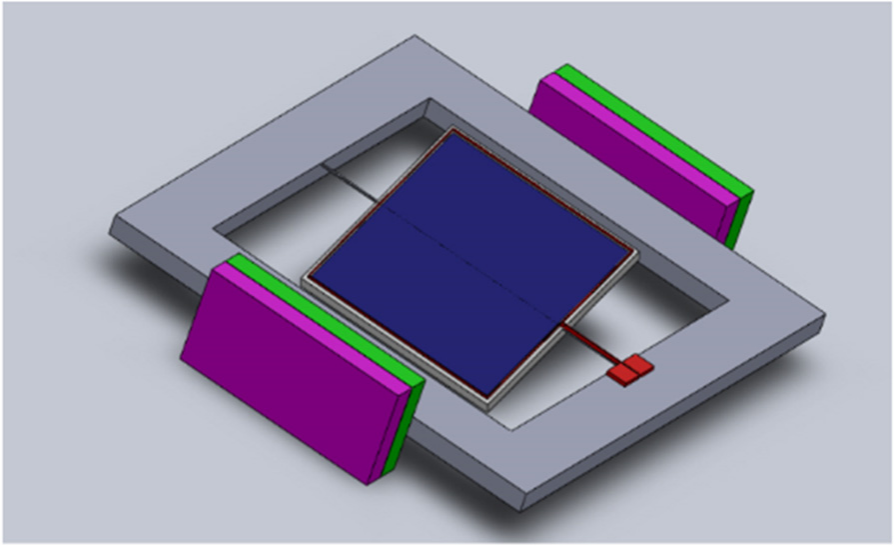
**Concept for a micromechanical integration of tilt principle by electromagnetic actuation.** Thick electroplated Cu lines are used to provide a current-controlled magnetic field which interacts with an external macromagnet.

**Figure 9:**
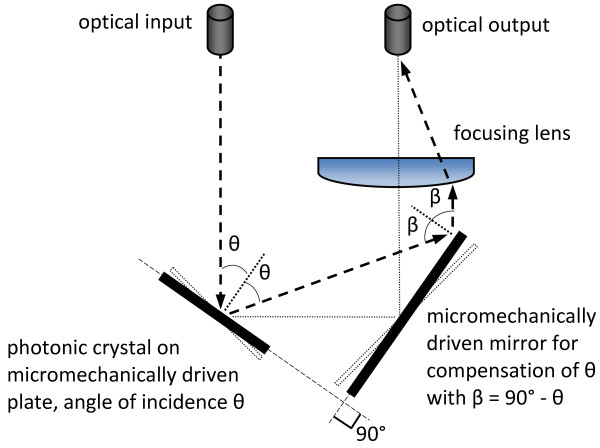
System integration of the developed TOF with two synchronously driven photonic crystal plates/mirrors.

Whereas electrostatic actuation with parallel charged capacitor plates for rotation is only feasible for small tilting angles, e.g., in milliradian range [15], electrostatic actuation using comb drives and electromagnetic actuator principles have been selected for further study. FEM simulations, analytical calculations, and fabrication process considerations have been performed (to be published separately). Based on the simulation, comb drive-based electrostatic actuation of 20° tilt angle will require around 70 V. On the other hand for the given demands, electromagnetic actuation has the capability for even larger tilt angles especially when using optimized square-shaped torsional beams for suspension of the porous Si photonic crystal. Additionally, fabrication is less complex. The concept of electromagnetic actuation is shown in Figure 8: an electromagnetically actuated photonic crystal reflector suspended by square-shaped torsional beams can provide tilt angles of up to ±20° at frequencies up to kHz even when using one metal layer only (electroplated 10-μm-thick Cu). Here the maximal possible current density in Cu lines and an outer magnetic field of 2 T were considered. A free-standing silicon plate with integrated porous silicon layers necessary for realization of this concept has been demonstrated before using a SOI process [16].In the final optical setup, the system is placed in a closed chamber with input and output orifices for gas or liquid and optical input/output fibers. In order to provide constant position of the optical input and output channels, a system with two synchronously actuated mirrors compensating tilt angle and focusing lens can be applied as shown in Figure 9.

## Conclusions

A novel MOEMS-based concept for tunable optical filter is presented. Combining fast micromechanical tilting and pore-filling of the porous-silicon-based photonic crystal, a tunable range of ±20% around the working wavelength of the TOF was realized. The tunability range for photonic crystals made out of low-doped p-type silicon was found to be wider than for photonic crystals made from high-doped p-type silicon. The feasibility of the concept was demonstrated experimentally. Experimental results confirmed the optical simulation results.

## Abbreviations

DBR: distributed Bragg reflector; DT: dual tunability; FPI: Fabry-Perot interferometer; MEMS: microelectromechanical system; MOEMS: microoptical electromechanical system; SEM: scanning electron microscope; SOI: silicon-on-insulator; TOF: tunable optical filter.

## Competing interests

The described tunable optical filter and the system concept have been submitted for a patent.

## Authors' contributions

UM, AK, and AI worked out the idea of dual tunability. UM supervised the master thesis work of IK and the work on development of the MOEMS tilting system for large deflection angles, and supplied the research activities with fruitful comments. IK conducted the tunability experiments and simulations as a part of his master thesis. AK led all the experimental and simulation activities and supervised the master thesis work of IK. AI developed the fabrication process for the photonic crystals and fabricated them, made initial experimental measurements on tilting, designed the optical system for the miniaturized concept, and made the final formatting and proof-reading of the paper. All authors wrote parts of the text and read and approved the final manuscript.
